# Robotic surgery in emergency general surgery: an overview of UK practice

**DOI:** 10.1186/s13017-025-00658-8

**Published:** 2025-11-18

**Authors:** Emily Francesca Smith, Michael Okocha

**Affiliations:** 1 University of Cambridge, Cambridge, UK; 2 Southmead Hospital, Bristol, UK

## Abstract

**Introduction:**

Laparoscopic surgery (LS) is well established in the United Kingdom (UK), while robotic surgery (RS) is increasingly adopted in elective practice. However, its role in emergency general surgery (EGS) remains undefined. This study provides the first national estimates of RS use in UK EGS, evaluating trends in utilisation, outcomes, and workforce capacity.

**Methods:**

Two rounds of Freedom of Information (FOI) requests were submitted to all NHS acute trusts and boards providing EGS services across Great Britain, covering 1 January 2019–1 January 2023 and 1 January 2023–1 January 2025. Data collected included robotic system ownership, numbers and types of robotic procedures, length of stay (LOS), complication rates, and staffing. LOS data were reported as mean ± standard deviation, with differences between approaches assessed using Welch’s t-tests.

**Results:**

Response rates were 83% (113/136 trusts, 2019–2023) and 65% (91/140 trusts, 2023–2025). Robotic availability increased from 36 to 91 systems nationally (147% rise), with the number of trusts performing emergency RS doubling (13 to 26). A total of 1816 emergency robotic procedures were performed in 2023–2025, alongside 9232 elective cases. Cholecystectomy was the most frequent emergency procedure, increasing almost sixfold (200 → 1396). Complications were infrequent, with device-related issues (*n* = 18) and tissue injury (n = 27) most common. LOS was consistently shorter for RS compared with open surgery across all procedures (all *p* < 0.01), with particularly marked reductions in cholecystectomy, Hartmann’s procedures, and small bowel resections. Comparisons with LS were procedure-specific: RS was shorter in cholecystectomy and hernia repair, equivalent in small bowel resection, and longer in appendectomy. Workforce capacity remained a limiting factor, with median in-hours trained staff unchanged (11 vs 10), but out-of-hours staff rising from 3 to 24 across all trusts.

**Conclusion:**

Robotic surgery in UK emergency general surgery is feasible, safe, and expanding, though utilisation continues to lag behind elective practice. RS offers clear LOS advantages over open surgery, with variable benefits compared to laparoscopy. Scaling adoption will require addressing cost, system heterogeneity, and the shortage of trained out-of-hours staff. National policy must prioritise training, standardised data collection, and equitable access to ensure safe integration of RS into routine emergency care.

## Introduction

Laparoscopic surgery (LS) is firmly established in the United Kingdom (UK), with a substantial body of literature comparing its outcomes to those of open surgery (OS) and robotic surgery (RS) in non-emergent settings. Numerous studies have demonstrated that RS often yields superior outcomes when compared to both LS and OS, particularly in terms of surgical precision and patient recovery [1–5]. The benefits of minimally invasive surgery (MIS), including LS and RS, over OS are well documented, with consistent evidence indicating reduced postoperative pain, shorter hospital stays, and lower risks of infection and mortality [6–8].

Despite the apparent rise in the adoption of RS in the UK, there remains a paucity of data regarding specialty-specific caseloads. The UK’s national database, the Hospital Episode Statistics (HES), has tracked laparoscopic procedures since 2000, but interpretation of these data is hampered by the lack of detailed outcome measures, specialty-specific information, and comprehensive differentiation between emergency and elective cases. Nevertheless, the data does indicate a significant increase in RS since it was first recorded in 2012/13, with 3,401 cases (compared to 207,105 LS cases). This number has grown annually, reaching 12,820 RS cases (LS 268,626) in 2016/17 and 37,751 RS cases (LS 266,008) by 2022/23 [9].

OS and LS remain the status quo for many emergency general surgery (EGS) procedures, and data suggests that OS continues to be the preferred method for a considerable proportion of EGS procedures [10]. Internationally, however, there has been a growing adoption of RS for specific EGS interventions, including cholecystectomies, colectomies, hiatal hernia repairs, and abdominal hernia repairs [11–13]. The World Society of Emergency Surgery published guidance on RS applications in EGS, highlighting the necessity of surgical expertise, adequately trained operating room staff, and strict patient selection criteria [14], Despite international advances, to our knowledge, a large-scale study to evaluate the use of RS in UK EGS has not been performed. A thorough investigation into RS’s role in EGS has the potential to support development of standards, training opportunities and to lay the foundation for future research.

## Methods

### Questionnaire design

The questionnaire was developed collaboratively by the two investigators and underwent iterative modifications based on feedback from the Freedom of Information (FOI) team and alignment with information governance protocols. Consistent with FOI guidelines, the design prioritised data acquisition within the constraints of limited time and reliance on non-surgical staff for data collection and analysis. The final instrument comprised eight questions, addressing key domains: all uses of robots in general surgery, its use in specific procedures, comparative length of stay, complication rates, and staffing metrics. Two time periods were studied (January 1st 2019-January 1st 2023 and January 1st 2023-January 1st 2025) in order to compare progression in robotic surgery operations. This study exclusively utilised aggregated, non-identifiable patient data to ensure compliance with ethical standards.

### FOI act requests

Two rounds of FOI requests were submitted to 122 NHS Trusts in England, seven Welsh Health Boards, and 14 Scottish Regional NHS Boards, identified as providing acute emergency general surgery services. Identification occurred through the NHS Service Directory, Welsh Government website, and NHS Research Scotland database. These were cross-referenced with the National Emergency Laparotomy Audit (NELA) and Emergency Laparotomy and Laparoscopic Scottish Audit (ELLSA) annual reports to ensure accuracy and comprehensiveness. For round 1, requests were sent between 26 August and 9 November 2023, with follow-ups from 9 December 2023 to 9 January 2024. Responses received by 6 April 2024 were included. For round 2, requests were sent between April 2025 and May 2025, with follow-ups from June 2025 until August 2025. Responses received by 31st July 2025 were included in this second round. Ethical approval was not required, as the Freedom of Information (FOI) Act 2000 permits public access to this data [15].

Under the FOI Act, public authorities are required to respond to requests within 20 working days unless the administrative cost exceeds £450, data availability is limited, or the information compromises personal privacy. The study analysed emergency general surgeries performed between 1 January 2019 and 1 January 2025, within two separate surveys, defined per the European Union of Medical Specialists [16].

### Analysis

Data were categorised by surgical approach—open, laparoscopic, and robotic—and included the number of robotic emergency procedures and semi-elective cases. Procedures were classified into cholecystectomy, appendectomy, hernia repair, and other with length of stay recorded for each approach. These procedures were selected for subgroup analysis based on their high frequency in emergency surgery [17].

Complications were categorised using previously reported classifications [18–20] including conversions, device-related issues, structural injuries, seromas, infections, leaks, and hernias. Conversions from minimally invasive to open surgery were included as intention-to-treat, as long as primary procedure had been coded for. Robotic to open conversions were included in complication totals. Data was also collected on robotic systems and the number of trained staff, with specific attention to out-of-hours staffing for robotic emergencies.

Per Section 40 of the FOI Act 2000, patient counts fewer than five were anonymised as “ < 5,” and for analysis, these values were estimated as 4.

Length of stay (LOS) data were supplied by NHS trusts as mean values with standard deviations, which limited the ability to perform non-parametric analyses. Differences in mean LOS between open, laparoscopic, and robotic approaches were therefore assessed using Welch’s two-sample t-tests, which account for unequal variances and group sizes. A *p*-value < 0.05 was considered statistically significant.

### Outcomes

The primary outcome was the number and types of robotic emergency general surgical operations occurring within Great Britain. Secondary outcomes looked at length of stay, out of hours staffing, complications and models of robots in use.

### Study covariates

Demographic data was unavailable, as the cost of identification, extraction, and collation exceeded the £450/18-h limit stipulated by Section 12 of the Freedom of Information Act.

## Results

### Responses

**Fig. 1:**
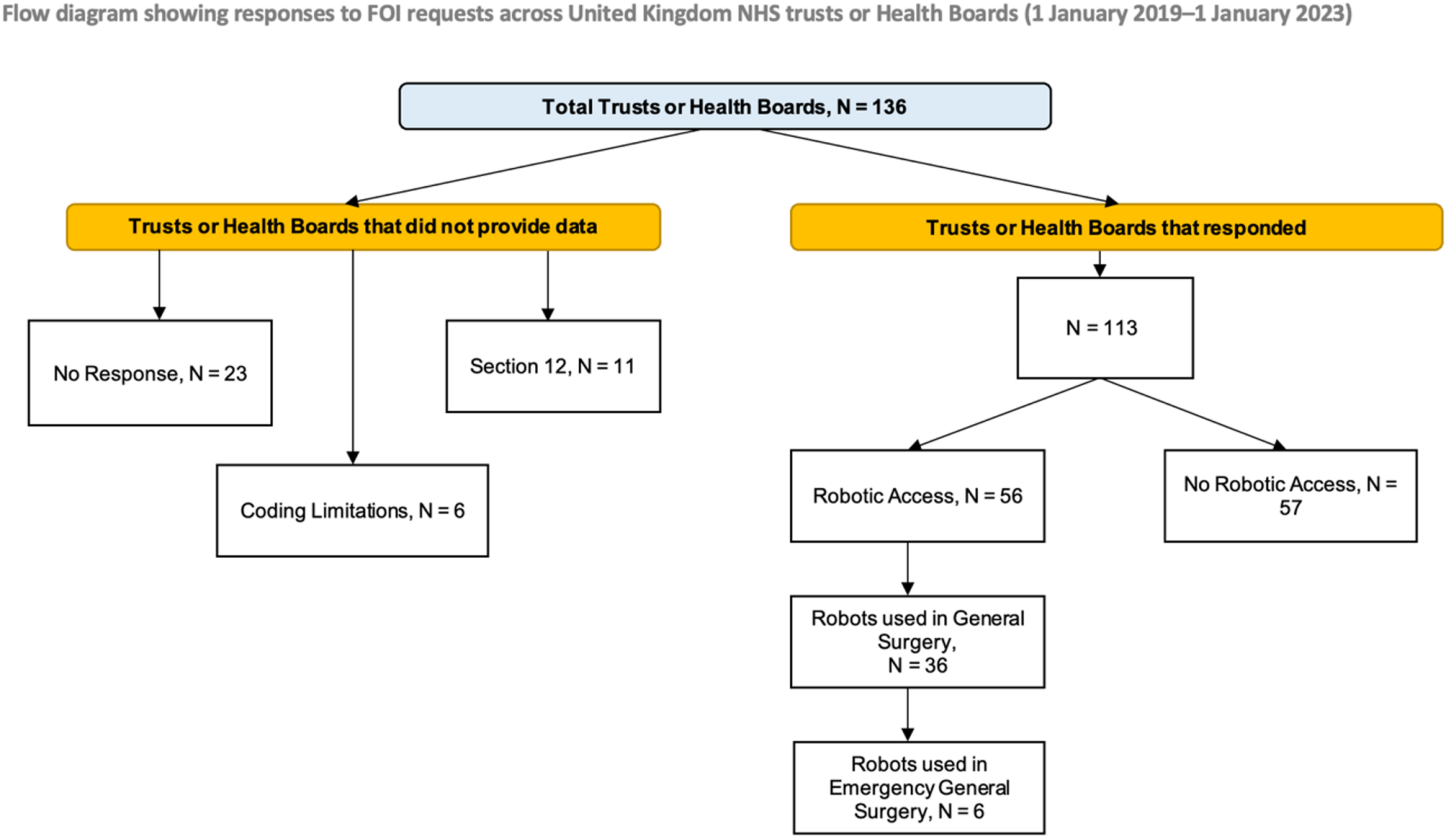
FOI PRISMA Flow diagram for period 1(1 January 2019–1 January 2023)

In response to the first questionnaire (1 January 2019–1 January 2023), 113 of 136 Acute Trusts provided data (83% response rate). Of responders 57 of 113 reported no access to robotic systems. Six trusts could not supply complete information owing to coding limitations, and eleven were exempt under Section 12 of the FOI Act (data retrieval costs > £450) (Fig. 1).

**Fig. 2:**
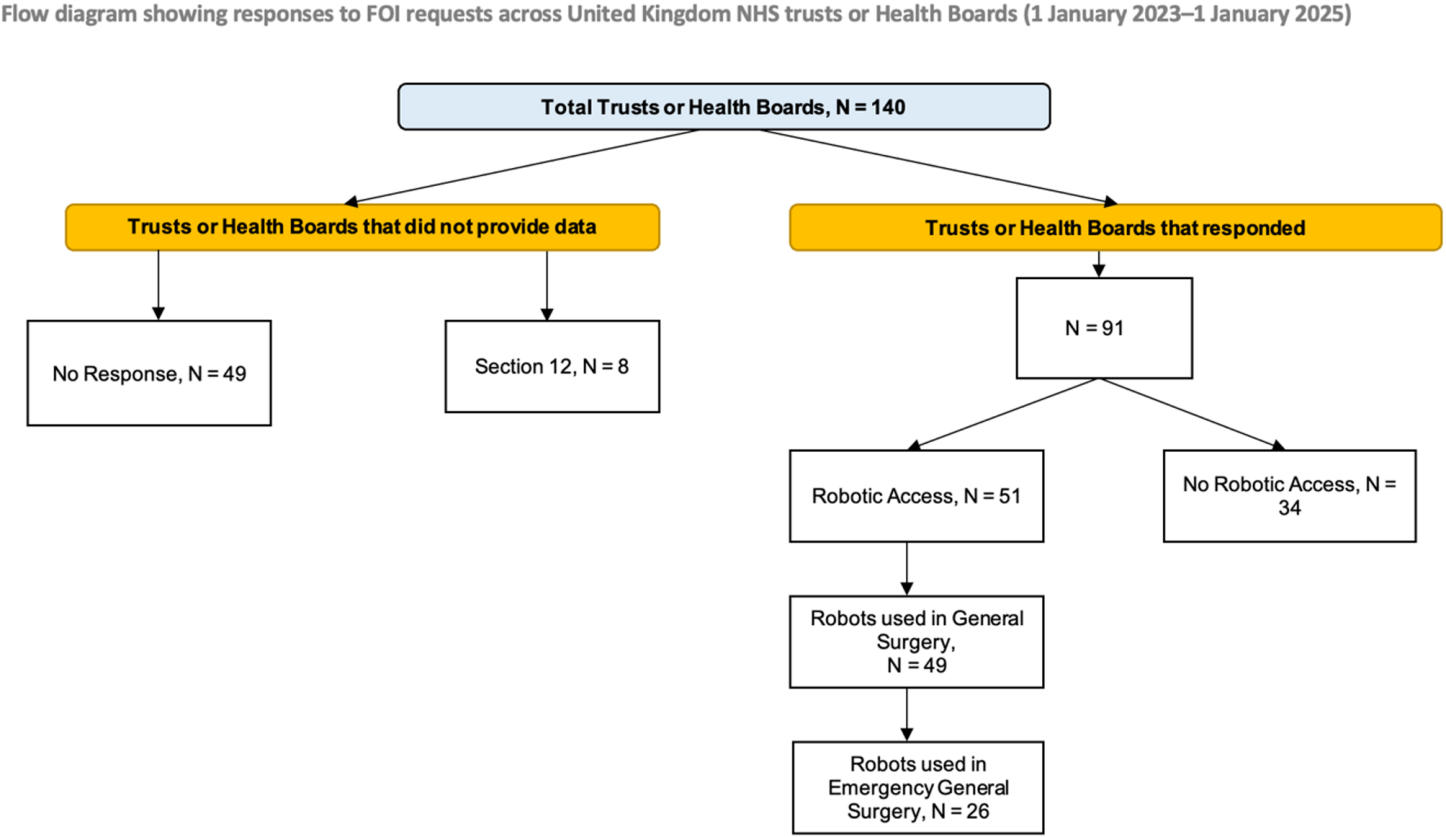
FOI PRISMA Flow diagram for period 2 (1 January 2023–1 January 2025)

For the period 1 January 2023–1 January 2025, 91 of 140 trusts responded (65% response rate). Eight cited Section 12 exemptions (data retrieval costs > £450). Thirty-two reported no access to robotic systems, while two were in the process of commissioning a robot. This represents a 43% reduction compared with 2019–2023 (Fig. 2).

### Robotic platforms

Between 2019 and 2023, 36 of 113 responding trusts reported access to a surgical robot for elective general surgery, while only 13 employed robotic systems in emergency settings. The Da Vinci platform predominated (29/36, 88%), with the remainder using the CMR Versius system (n = 7). The mean number of robots per trust was one, giving a national total of 36 systems.

By 2023–2025, access had expanded: 51 trusts reported owning a surgical robot, of which 49 used them in general surgery and 26 in emergency general surgery (Table 1). This represents a 42% increase in ownership (36 to 51 trusts) and a 100% increase in emergency use (13 to 26 trusts). The total number of robots rose from 36 to 91 (147% increase), with an average of 1.93 per general surgery trust. Three trusts did not specify the platform in use. The Da Vinci system remained dominant, accounting for 68 robots (X and Xi models combined). Across both time periods, the Da Vinci platform accounted for over twice as many general surgical procedures as the CMR system alone. Trusts with multiple robotic platforms were unable to specify which model was used for each case (Table 2).

**Table 1:** Frequency of different robotic systems for all specialties

	Robot type	Number of trusts
January 1st 2019-January 1st 2023	DaVinci System	29
	CMR Versius	7
January 1st 2023-January 1st 2025	DaVinci System	68
	CMR Versius	9
	Renishaw Neuromate	2
	Medtronic Hugo	3
	Medtronic Mazor	1
	Intuitive Ion	2
	Mitsubishi Electric RH-FRH	3
	MAKO	3

**Table 2:** Showing the number of emergency and total number of general surgical procedures perfromed by each robot model across the two time periods 2019–23 and 2023–25

Years	Robot configuration	Emergency general surgery procedures	Total general surgery procedures
2019–23	Da Vinci	102	8112
2019–23	CMR / Versius	1	3111
2019–23	Da Vinci + CMR / Versius	2	1520
2023–25	CMR Versius	9	299
2023–25	Da Vinci X/XI	1,780	7087
2023–25	CMR + Da Vinci	11	438
2023–25	Da Vinci + HUGO	0	127
2023–25	Da Vinci + CMR Versius + Medtronic Hugo	0	404

Over the same period, the diversity of robotic systems increased. In 2019–2023, three platforms were reported in use across Great Britain: Da Vinci, CMR Versius, and MAKO (restricted to Trauma and Orthopaedics). By 2023–2025, this had expanded to eight distinct systems. The increase in both numbers and variety reflects growing investment in robotic surgery within the NHS. These findings are consistent with data reported by Marchegiani et al. [21] on robotic system characteristics and costs in UK hospital trusts (Table 3).

**Table 3:** Characteristics and costs of robotic systems used in general surgery across NHS trusts in Great Britain as demonstrated in Marchegiani et al. [21]

Company	Robot	Origin	Architecture	Arms	Instruments’ reusability	Cost of purchase (GBP £)
Cambridge Medical Robotics	Versius ®	UK	Modular	3	Reusables	£1.2-£1.5 million
Intuitive surgical	Da Vinci SP ®	US	Single Port	3	Reusables	£0.5–2.5 Million (Eckhoff et al., 2023)

### Robotic procedures performed

In the first period (1 January 2019–1 January 2023), 13 trusts performed robotic emergency general surgery. All recorded procedures—cholecystectomy, appendectomy, diagnostic robotic laparoscopy, intra-abdominal abscess drainage, and hernia repair—had been attempted at least once. Cholecystectomy was most frequent (n = 200), followed by hernia repair (n = 42) and appendectomy (n = 28). Reported complications were rare, with only one which was postoperative bowel obstruction.

In the second period (1 January 2023–1 January 2025), 49 trusts reported robotic emergency general surgery activity. Cholecystectomy again predominated (n = 1396), representing an almost 600% increase (200 → 1396) from the earlier period. Across all procedure types, numbers rose substantially, with 1816 robotic emergency operations performed. There were coding discrepancies in which conversions to laparotomy were recorded as “robotic laparotomies,” and some adhesiolysis cases were coded as “robotic adhesiolysis.” Additionally intra-abdominal abscess drainage (put the H200 code etc. here) was recorded as primary procedure instead of a secondary. In all instances, the primary procedure could not be identified; these cases were therefore excluded from detailed analysis and are presented as “other”. In total, 9232 robotic general surgery procedures were undertaken across both elective and emergency settings, equating to an average of 196.4 per trust (Table 4).

**Table 4:** Number of emergency robotic, laparoscopic and open surgeries taking place categorised by type of operation

	Operation type	Robotic approach	Laparoscopic approach	Open approach
January 1st 2019-January 1st 2023	Cholecystectomy	200	20,217	739
	Appendectomy	28	1883	17,951
	Hernia Repair	42	4214	17,226
	Other	6	139	9068
January 1st 2023-January 1st 2025	Cholecystectomy	1396	45,140	5918
	Appendectomy	139	27,598	3758
	Hernia Repair	1064	10,057	47,981
	Hartmann’s	572	1265	3211
	Small bowel resection	72	579	4657
	Other	2706	12,729	14,491

### Complications

Complications reported in 2023–2025 included conversions (n = 33; mean 2.1 per trust), device-related problems (n = 18; mean 1.1), injury to surrounding tissues (n = 27; mean 1.7), seromas (n = 4; mean 0.3), infections (n = 43; mean 2.7), leakages (n = 47; mean 3.0), and hernias (n = 5; mean 0.3).

### Length of stay

Mean length of stay (LOS) varied by procedure and operative approach across both study periods. In 2019–2023, robotic cholecystectomy was associated with the shortest LOS at 1.1 ± 1.2 days, compared with 3.0 ± 2.5 for laparoscopic and 10.6 ± 6.1 for open surgery. For appendectomy, LOS was lowest with laparoscopy (3.7 ± 5.1 days), followed by robotic (4.5 ± 3.6) and open (6.9 ± 2.3). Hernia repair showed broadly similar values, with LOS of 3.9 ± 3.5 (open), 6.3 ± 14.4 (laparoscopic), and 4.1 ± 3.6 (robotic).

In 2023–2025, robotic cholecystectomy again had the shortest LOS at 1.6 ± 2.6 days, compared with 2.1 ± 1.8 for laparoscopic and 8.7 ± 4.6 for open. Appendectomy LOS remained lowest for laparoscopy (3.1 ± 1.5), but was longer for robotic (5.0 ± 10.0) and open (11.1 ± 17.7). Hernia repair was comparable across approaches, with LOS ranging from 2.6 ± 2.5 (robotic) to 3.0 ± 2.9 (laparoscopic) and 2.8 ± 2.6 (open). For procedures newly captured in this period, robotic Hartmann’s (11.5 ± 10.7 days) and small bowel resection (11.3 ± 8.7) were both associated with shorter LOS than laparoscopic (14.3 ± 8.0 and 13.2 ± 9.5, respectively) and open approaches (17.3 ± 6.3 and 21.5 ± 13.0).

Across both time periods, robotic approaches were consistently associated with shorter LOS than open surgery for all procedures analysed (all *p* < 0.01) (Table 5). For cholecystectomy, robotic LOS was significantly lower than both laparoscopic and open (*p* < 0.0001). In appendectomy, laparoscopic LOS remained the shortest; robotic was similar to laparoscopy in 2019–2023 (*p* = 0.26) but longer in 2023–2025 (*p* = 0.027). For hernia repair, robotic LOS was shorter than laparoscopic in both periods (*p* < 0.001), with no difference compared to open in 2019–2023 but significantly shorter in 2023–2025 (*p* = 0.01). Among procedures captured only in 2023–2025, robotic Hartmann’s and small bowel resections both demonstrated significantly shorter LOS compared to open surgery (*p* < 0.0001), with robotic Hartmann’s also shorter than laparoscopic (*p* < 0.0001).

**Table 5:** Pairwise Welch’s t-tests comparing mean length of stay (LOS, days) between open, laparoscopic, and robotic approaches for emergency general surgical procedures across both study periods

Procedure (Period)	Comparison	t-stat	*p*-value	Interpretation
Cholecystectomy (2019–23)	RS vs LS	-21.9	< 0.0001	RS shorter LOS
	RS vs OS	-39.6	< 0.0001	RS shorter LOS
	LS vs OS	-33.8	< 0.0001	LS shorter LOS
Appendectomy (2019–23)	RS vs LS	1.16	0.26	NS difference
	RS vs OS	-3.53	0.0015	RS shorter LOS
	LS vs OS	-26.9	< 0.0001	LS shorter LOS
Hernia Repair (2019–23)	RS vs LS	-3.68	0.0005	RS shorter LOS
	RS vs OS	0.36	0.72	NS difference
	LS vs OS	10.7	< 0.0001	LS longer LOS
Cholecystectomy (2023–25)	RS vs LS	-7.13	< 0.0001	RS shorter LOS
	RS vs OS	-77.1	< 0.0001	RS shorter LOS
	LS vs OS	-108.4	< 0.0001	LS shorter LOS
Appendectomy (2023–25)	RS vs LS	2.24	0.027	RS longer LOS
	RS vs OS	-6.81	< 0.0001	RS shorter LOS
	LS vs OS	-27.7	< 0.0001	LS shorter LOS
Hernia Repair (2023–25)	RS vs LS	-4.88	< 0.0001	RS shorter LOS
	RS vs OS	-2.58	0.010	RS shorter LOS
	LS vs OS	6.40	< 0.0001	LS longer LOS
Hartmann’s (2023–25)	RS vs LS	-5.59	< 0.0001	RS shorter LOS
	RS vs OS	-12.6	< 0.0001	RS shorter LOS
	LS vs OS	-12.0	< 0.0001	LS shorter LOS
Small Bowel (2023–25)	RS vs LS	-1.73	0.087	NS difference
	RS vs OS	-9.78	< 0.0001	RS shorter LOS
	LS vs OS	-18.9	< 0.0001	LS shorter LOS

### Staff trained

For the time period of January 1st 2019-January 1st 2023, the median number of staff trained to assist with robotic general surgery during standard hours was 11 (range 2–75) among the 13 trusts performing both elective and emergency robotic procedures. However, out-of-hours coverage was limited, with only three trained staff across all 13 trusts (range 0–3). In comparison, trusts performing only elective robotic surgery (n = 20) reported a median of 11.5 trained staff in-hours (range 2–24) and 10 out-of-hours staff across all 20 trusts (range 0–4).

For the time period of January 1st 2023-January 1st 2025 the median number of staff trained to assist with robotic general surgery during normal working hours was 10 (range 3–93). In total across all trusts there were 24 staff trained to assist in out of hours robotic surgeries. This data is limited by the 27 trusts that could not provide an answer to this question on the survey due to Sections 12 or 17.

## Discussion

This study provides the first national estimates of robotic surgery (RS) utilisation in emergency general surgery (EGS) across the UK. While RS adoption has accelerated in elective practice, uptake in EGS remains limited, with only 26 of 91 responding trusts reporting use by 2025. Elective RS volumes rose to 9232 procedures in 2023–2025 (from a few hundred in 2019–2023), whereas emergency RS totalled 1816 procedures, highlighting a slower trajectory of integration.

Our findings demonstrate the feasibility of robotic-assisted emergency procedures, including cholecystectomy, hernia repair, and appendectomy, with low complication rates consistent with international literature (11–13). The most frequent adverse events in 2023–2025 were device-related problems (n = 18) and tissue injuries (n = 27), underscoring the importance of system refinement, robust maintenance, and structured training as case volumes expand.

Robotic availability also increased markedly between 2019 and 2025: the number of trusts with a robot rose by 42%, total systems increased by 147%, and the mean number per trust nearly doubled (1.0 → 1.93), reflecting significant investment in access and capacity for general surgery. A sixfold rise in robotic cholecystectomy indicates its growing acceptance as a reproducible index procedure.

Analysis of length of stay (LOS) data showed that RS was consistently associated with shorter admissions than open surgery across all procedures, with particularly marked reductions in cholecystectomy, Hartmann’s procedure, and small bowel resection. Comparisons with laparoscopy were procedure-specific: LOS was shorter for RS in cholecystectomy and hernia repair, equivalent in small bowel resection, and longer in appendectomy. These findings suggest that the benefits of RS over laparoscopic surgery are not universal and may depend on case selection and operative context.

The expansion of robotic surgery from 2019 to 2025 also carries important workforce implications. While the median number of in-hours trained staff per trust has remained stable (median 11 to 10), the eightfold rise in out-of-hours trained staff (total 3 to 24) suggests an increasing recognition of the need for broader service provision. Yet, absolute numbers remain small, and workforce capacity continues to limit the feasibility of emergency robotic surgery. Addressing these gaps will require coordinated national strategies for training, credentialing, and rota planning.

Cost remains a persistent barrier to widespread adoption. System heterogeneity—including differences in architecture, instrument reusability, and number of operative arms—complicates procurement and benchmarking. Although the Da Vinci platform remains dominant, diversification of the market provides an opportunity for cost competition. Formal evaluation of cost-effectiveness, both in terms of capital investment and long-term patient outcomes, will be essential to justify scaling within the NHS.

One key advantage of RS lies in its potential to improve outcomes in selected patients, however, this study demonstrates persistent barriers to broader adoption in EGS, most notably financial constraints, limited system availability, and critically, the shortage of trained staff during out-of-hours periods. While the median number of in-hours trained staff per trust was 11, this fell to a median of 0 out-of-hours, not reflecting the unpredictable nature of emergency care and highlighting the workforce challenges it entails. This discrepancy represents a missed opportunity for training and utilisation of robotic systems in emergent settings, an issue of particular importance for future generations of surgeons [19, 22].

The trajectory of RS adoption within the NHS suggests movement towards mainstream use in both elective and, increasingly, emergency practice. To support this transition, national policy must prioritise equitable access, structured workforce training frameworks, and standardised data collection to ensure safe and efficient scaling. Nonetheless, the cost of robotic platforms, skill transferability and variation between systems remain key considerations. Further research into cost-effectiveness, long-term outcomes, and procedure-specific benefits will be essential before RS can be considered the standard of care across general surgery.

### Limitations

This study was constrained by incomplete data from non-responding trusts and by the inherent limitations of Freedom of Information (FOI) requests, including Section 12 exemptions that restricted access to certain datasets. The absence of patient-level data and information on case selection further limited the ability to evaluate outcomes and directly compare robotic, laparoscopic, and open approaches.

Despite these challenges, the findings underscore the need for sustained investment in training and infrastructure to support the safe expansion of robotic surgery in EGS. In particular, addressing the shortage of trained out-of-hours staff is critical. Structured training pathways—incorporating simulation, mentorship in high-volume centres, and inter-hospital collaborations—could enhance both accessibility and implementation, supporting the transition of robotic systems into routine emergency care.

## Conclusion

The UK has a clear opportunity to expand the integration of robotic surgery into emergency general surgery. By addressing current barriers—most notably the shortage of trained staff and disparities in infrastructure—there is substantial potential to improve both patient care and surgical training. Prioritising dedicated robotic training programmes will be essential to prepare the next generation of surgeons for modern practice. Strategic investment in training and capacity building will not only accelerate adoption but also strengthen the UK’s position in global surgical innovation.

## Data Availability

The datasets generated during the current study are available from the corresponding author on reasonable request.
